# Plant Antioxidants for Food Safety and Quality: Exploring New Trends of Research

**DOI:** 10.3390/antiox10060972

**Published:** 2021-06-17

**Authors:** Martina Loi, Costantino Paciolla

**Affiliations:** 1 Institute of Sciences of Food Production, National Research Council, Via Amendola 122/O, 70126 Bari, Italy; martina.loi@ispa.cnr.it; 2 Department of Biology, University of Bari Aldo Moro, Via E. Orabona 4, 70125 Bari, Italy

Antioxidants are an heterogeneous group of compounds able to counteract cell oxidation by acting as reducing agents, as free radical scavengers, and quenchers of radical species and other pro-oxidants, such as metals [1]. Plants are a remarkable source of antioxidants, such as polyphenols (phenolic acids, lignans, stilbenes, and flavonoids such as anthocyanins,), carotenoids (xanthophylls and carotenes), and vitamins (vitamin E and C). In plants, such compounds possess different functions, including signaling; defense towards insects, bacteria, and fungi; prevention of oxidative damage; and free-radical scavenging [2]. They also confer color, aroma and taste to fruits, vegetables, and the derived processed products. Plant antioxidants play an important role in the human diet. Their consumption has been correlated with a lower risk of cardiovascular and neurodegenerative diseases and cancer, lower inflammation levels, improvement in cognitive functions, mitigation of the detrimental effects of reactive oxygen species, and ageing [3,4,5].

Owing to their antioxidant and antimicrobial characteristics, many efforts are devoted to increase their content in plants in the field [6], to prevent their loss during food processing and storage, to extract them from waste or by-products, and to exploit their antioxidant potential in functional foods or supplements. In foods, higher antioxidant content promotes the oxidative stability, especially of foods containing lipids [7].

This Special Issue aimed to collect the most recent outcomes of plant antioxidants research, with particular regards to all aspects of antioxidant application and the production of healthier and safer foods.

Food processing and storage appear to be two important fields of intervention to maintain high levels of phenols, flavonoids, and tannins, and promote the generation of antioxidant peptides. Thermal treatments are usually used to ensure microbial safety and to inactivate undesired enzymatic activities [8]. At high temperatures chemical reactions responsible for the generation of taste and aroma take place, proteins denature, and lipids oxidize. Antioxidant compounds are generally sensitive to heat, and their biological activity is reduced. During storage, oxidation of antioxidants is promoted by oxidative enzymes, pH value, light, and high temperature [7].

The current trends of research explore novel biotechnological protocols to reduce the loss of antioxidants, and investigate the conventional methods to assess their impact on their content and bioaccessibility. In addition, the effects of added-antioxidants on thermal treatments and the storage of different food products is another important topic. Predictive modelling of antioxidant and protein oxidation, with the support of artificial neural networks, is also emerging as a powerful tool to monitor quality changes.

Three different studies evaluated the impact of novel and conventional processing techniques on strawberry [9], coffee [10], and hemp matrices [11].

Stübler and colleagues [8] investigated the stability and bioaccessibility of anthocyanins in strawberry puree and in a strawberry puree—kale juice mix during refrigerated storage. They also compared the traditional thermal stabilization to two different non-thermal treatments, pulsed electric fields (PEF), and high-pressure processing (HPP). Anthocyanin degradation was more pronounced in the strawberry-kale mix, possibly due to enzymatic oxidation, and HPP was the best treatment to preserve them, followed by PEF, and then the thermal treatment. On the contrary, the thermally and PEF-treated samples exhibited the highest gastric bioaccessibility rate. Thus, HPP and PEF proved to be efficient methods to preserve the antioxidant content of strawberry and its bioaccessibility.

Haile and colleagues [10] compared the antioxidant and volatile compounds of coffee obtained by different processing methods, i.e., the digestive bioprocessing method used to obtain the elephant dung coffee, and two conventional processing methods, wet and dry. They found that the digestive processing method lead to higher phenol, flavonoid, and tannin contents, while there were similar outcomes in terms of volatile compounds.

Pontonio and colleagues [11] studied a novel enzymatic and microbial bioprocessing to improve the antioxidant, nutritional, and functional properties of hemp (*Cannabis sativa* L.). Hemp bioprocessing consisted of a combined enzymatic treatment with commercial food grade enzymes and ad hoc selected lactic acid bacteria. The antioxidant potential of hemp was significantly improved through intense proteolysis followed by release of antioxidants peptides and bound phenolics. The antioxidant properties increased when tested in vitro on the human keratinocyte cell line. Due to proteolysis, hemp-food protein quality available for digestion and assimilation was also improved.

The effect of added antioxidants to reduce food oxidation and maintain high quality during the shelf life is an important matter of research. Three different studies focused on this topic, analyzing lipid and protein oxidation in meat, namely beef [12], pork [13], and lamb [14]. One study focused on lipid oxidation in a vegetable oil [15]. Two also developed predictive models to monitor oxidation during storage. Indeed, lipid oxidation is recognized as the main factor responsible for the decrease in the sensory and nutritional quality of meat, especially in minced meat and during storage [12]. Meat protein oxidation is also an important parameter to consider, as it may trigger lipid oxidation, decrease digestibility, and lead to the loss of essential amino acids.

Kaczmarek and colleagues [12] developed and compared the predictive models of lipid oxidation in minced raw beef meat supplemented with different plant extracts during storage. Lipid oxidation increased significantly with increasing storage time and temperature, but were less pronounced with the addition of the plant extracts, with clove, allspice, and bay leaf being the most effective. Lipid oxidation was also modelled with an Arrhenius model to allow the monitoring of oxidative changes in ground meat under different time and temperature conditions.

Muzolf-Panek and colleagues [13] evaluated the effect of 13 plant extracts in decreasing protein oxidation in raw ground pork during storage at different temperatures. They found that oregano was the best extract in preventing protein oxidation. Interestingly, no correlation was observed between the radical scavenging activity of the extracts and their activity in meat, suggesting that there may be other mechanisms underlying the protective effects in the matrix. Predictive modelling and validation was also performed for the temperature dependence oxidation by the Arrhenius and log-logistic models.

Wang and colleagues [14] evaluated the impact of lycopene dietary supplementation in lambs to improve the quality of their meat. Lycopene supplementation (200 mg kg^−1^) in lamb diet increased the antioxidant capacity of postmortem meat and reduced drip loss during storage, likely due to the regulation of the expression of oxidases, metabolic enzymes, calcium channels, and structural proteins. The lipid oxidative stability was also promoted.

Flores and colleagues [15] studied avocado oil supplementation with maqui tree leaf extracts, an endemic Chilean fruit renowned for its antioxidant properties. Maqui extracts obtained with different solvents proved to be beneficial in reducing the thermal oxidation (exposure at 120 °C for 386 h) of avocado oil. The best results were obtained with the methanolic extract. Despite the promising results, the authors underline that before being used as an additive, further studies focused on the possible toxicity and hormetic role are needed.

Supplementation of antioxidants can be performed in various foods, not only to promote its oxidative stability, but also to obtain healthier and functional foods. Indeed, the study of Cedeño-Pinos and colleagues [16] focused on the supplementation of jelly candies made with fructan fibers and stevia with rosemary extract. The addition of the aqueous extracts derived from rosemary distillation increased the polyphenol content and the antioxidant capacity. In addition, polyphenols were retained after cooking, acting as secondary antioxidants and provided higher oxidative stability.

The changes in food quality induced by the application of natural preservatives, like plant antioxidants, that were the focus of these studies can be investigated with different technological approaches. One is spectroscopy, reviewed in depth by Hassaun and colleagues [17]. Spectroscopic techniques, such as Fourier-transform infrared spectroscopy, Raman spectroscopy, proton nuclear magnetic resonance, and fluorescence spectroscopy have great potential, as they are non-destructive, versatile, can be non-targeted, and provide valuable information on different food components. The only drawback is represented by the limits of the implementation of such technologies in industrial production systems.

Antioxidants can be also used as part of a packaging material in polymer and biopolymer formulations. They represent a cost effective and green alternative to synthetic antioxidants, with lower safety and environmental issues. This is the case of the study of Rojas-Lema and colleagues [18] who studied the application of naringin, gallic acid, caffeic acid, and quercetin for the manufacture of a bio-based high-density polyethylene (bio-HDPE). The inclusion of such antioxidants did not affect the mechanical properties of the polymer, but improved its thermo-oxidative stability, the onset degradation temperature, and showed a high antioxidant activity. Amongst all, quercetin proved to be the best antioxidant, with a promising future in the packaging sector.

Among the novel technologies used to improve the antioxidant profile of fruits and vegetables, light emitting diodes (LEDs) is one of the most promising. LEDs show unique spectra wavelengths, high luminous efficacy, lower operational cost, a lack of radiant heat, and longer lifespan compared to the traditional light systems.

As reviewed by Loi and colleagues [19], light quality and intensity can be perceived by plants by different photoreceptors, and elicit a wide range of biochemical changes. LEDs can stimulate the expression of genes related to the enzymes involved in ascorbate-glutathione cycle, and in the polyphenol, carotenoid, and glucosinolate synthesis. LED response is species and cultivar specific, thus it is important to carefully evaluate their effects for each vegetable of interest. LEDs are versatile, as they can be applied as supplemental light in the fields cultivated during the dark season [20], or during post-harvest, to increase the shelf life and phytonutrient content of food [21].

In the research of Palmitessa and colleagues [22], the effects of supplemental LED light on the physio-chemical parameters in tomato fruit was assessed. They found that fruits grown under LEDs had 3% more dry weight, 15% more total soluble solids, 16% higher titratable acidity, and 15% more vitamin C content than those grown only under natural light. No difference in lycopene content was observed. Therefore, through LEDs, they were able to produce more food maintaining high quality production.

The interest in specific antioxidants is still high in the scientific community. Two reviews focused on two different antioxidants, namely hydroxytyrosol and anthocyanins.

Hydroxytyrosol is one of the main olive antioxidants, recognized by EFSA for its role in reducing the risk of cardiovascular diseases [23]. Silva and colleagues reviewed its application as a functional ingredient in food and as a dietary supplement [24]. The real potential of hydroxytyrosol still has to be elucidated, as, to date, the clinical studies have only considered hydroxytyrosol in olive oil, and not as a single compound. Due to the bitter taste that it confers to food, and the expensive procedures needed for its extraction, further comprehensive studies need to be performed to allow the complete valorization and exploitation of such important antioxidant.

Yamuangmorn and colleagues [25] reviewed the potential of high-anthocyanin purple rice as a functional ingredient in human health. In particular, they followed rice anthocyanin from its synthesis and accumulation in the plant, to its fate in the processed products, and the health benefits in humans. The authors highlight the need of further studies on the bioavailability and metabolites of anthocyanins, as well as the potentialities of anthocyanin extraction from rice by products.

These reviews highlighted that there is increasing interest on the effects of plant antioxidants on human health and, in particular, on the characterization of the biological activity of novel antioxidants or plant extracts. Efenberger-Szmechtyk and colleagues [26] studied the biological effects of *Aronia melanocarpa*, *Cornus mas*, and *Chaenomeles superba* leaf extracts, which were previously used to extend the shelf life and improve the health benefits of meat products. The anti-cancer activity of these extracts was assessed in Caco-2 cells. The authors measured the antiproliferative activity and the morphological changes induced by the extracts. When low concentrations were used, the antioxidant properties stimulated DNA repair in Caco-2 cells, whereas at higher concentrations the extracts acted as pro-oxidant, leading to DNA damage. Further research is still required; nonetheless this preliminary study shed light into the possible use of these extracts as anticancer agents.

The papers published both in forms of original contribution and up-to-date literature reviews provide a comprehensive and interesting overview of the most recent and innovative trends of research in the field of plant antioxidants for food safety and quality. Nonetheless, further research is still needed to continue exploring the immense potential of plant antioxidants.
